# CHRNA3⁺ nociceptors prime the cutaneous sensory interface to enhance electroacupuncture analgesia

**DOI:** 10.1186/s13020-026-01425-w

**Published:** 2026-05-19

**Authors:** Wenjie Xu, Dingdan Zhang, Yuanwei Tang, Ying Wang, Zijie Wang, Hanqing Xi, Xinyan Gao, Bing Zhu, Xiang Cui

**Affiliations:** 1 Department of Physiology, Institute of Acupuncture and Moxibustion, China Academy of Chinese Medical Sciences, Beijing, 100700 China; 2 Shenzhen Bao’an Traditional Chinese Medicine Hospital, Guangzhou University of Chinese Medicine, Shenzhen, Guangdong Province 518100, China; 3 Department of Hematology and Oncology, Dongzhimen Hospital, Beijing University of Chinese Medicine, Beijing, 100700 China; 4 Shaanxi Provincial Hospital of Traditional Chinese Medicine, Xi’an, Shaanxi Province 710082, China; 5 Northeast Asia Research Institute of Traditional Chinese Medicine, Changchun University of Chinese Medicine, Changchun, Jilin Province 130117 China; 6 Hubei University of Chinese Medicine, Wuhan, Hubei Province 430065, China

**Keywords:** Acupoint sensitization, Mechanoinsensitive nociceptors, Silent nociceptors, Electroacupuncture

## Abstract

**Background:**

Recent evidence determined that acupoints frequently overlap with regions of referred somatic hypersensitivity induced by visceral disease, a phenomenon known as acupoint sensitization. This state is typically characterized by sensory hypersensitivity and functional enhancement, often accompanied by superior therapeutic outcomes following acupuncture. However, the neurobiological mechanisms that prime acupoints for enhanced responsiveness remain poorly understood.

**Methods:**

Using a rat model of TNBS intracolonic injection-induced colitis and relative acupoint sensitization, we investigated the role of CHRNA3⁺ mechanoinsensitive nociceptors (MINs), a subclass of silent C-fiber neurons, in modulating the acupoint sensory interface and electroacupuncture (EA) responsiveness. Behavior tests, neuroanatomical tracing, *in situ* hybridization, pharmacological blockade, and chemogenetic silencing were employed to assess the involvement of CHRNA3⁺ MINs in the onset of acupoint sensitization. Colonic distension-evoked visceral motor reflex and spinal local field potential recording were utilized to evaluate the contribution of CHRNA3⁺ MINs in the acupuncture-induced analgesic effect.

**Results:**

We found that CHRNA3⁺ MINs are primarily C nociceptors co-expressing TrkA, pERK, and PIEZO2, innervating both the colon and lumbar skin (BL25 acupoint region) via axonal bifurcation. Colitis significantly activated CHRNA3⁺ nociceptors via the NGF–TrkA/pERK/PIEZO2 pathway, converting them from mechanoinsensitive to mechanosensitive. This activation correlated with increased plasma extravasation and mechanical allodynia at BL25. Pharmacological inhibition of CHRNA3⁺ MINs via pERK blockade or chemogenetic silencing alleviated mechanical hypersensitivity of BL25 and attenuated the analgesic effect of EA on both visceral pain and spinal sensitization.

**Conclusion:**

Our findings reveal that CHRNA3⁺ silent nociceptors dynamically prime and reshape the sensory interface of the colitis-induced sensitized acupoint BL25, subsequently facilitating both pathological hypersensitivity and therapeutic responsiveness by heightening mechano-responsiveness of the acupoint. This study establishes a mechanistic link between visceral dysfunction, acupoint functional plasticity, and EA-induced therapeutic neuromodulation.

**Supplementary Information:**

The online version contains supplementary material available at 10.1186/s13020-026-01425-w.

## Introduction

Acupuncture has been practiced for millennia, yet the anatomical basis of acupoints remains elusive. Numerous studies have compared acupoints to adjacent non-acupoint regions, reporting subtle differences in sensory nerves [1], blood vessels, lymphatic vessels [2], connective tissues [3], tissue space areas [4], and mast cells [5]. The absence of a striking anatomical signature raises a critical question: are acupoints fixed landmarks or dynamic, state-dependent sensory interfaces? Two longstanding challenges in acupuncture research compound this question. First, sham or “non-acupoint” controls often elicit effects comparable to true acupoints, undermining their validity. Second, the “wide-pan-acupoint” phenomenon, therapeutic benefit from needling outside traditional points, suggests that acupoint efficacy depends more on the local sensory state than on precise location. Together, these observations argue for reframing acupoints as plastic sensory interfaces, reshaped by disease, particularly visceral diseases, rather than immutable anatomical sites. Clinically, needling hypersensitive regions, such as Ashi and trigger points, consistently yield promising therapeutic outcomes, highlighting the potential role of sensory plasticity in acupoint function.

Acupoint sensitization is a recently characterized functional state in which somatic regions corresponding to acupoints exhibit sensory abnormalities under pathological conditions such as visceral disease. This state is defined by two key features: sensory hypersensitivity (e.g., mechanical allodynia) and heightened responsiveness to therapeutic stimuli like acupuncture [6–13]. Although these sensitized areas frequently overlap with regions of visceral referred pain [6–13], the core concept of acupoint sensitization emphasizes the resulting sensory abnormality and functional plasticity, which optimizes these sites for receiving and transducing therapeutic stimuli. Neurogenic inflammation is a key pathological driver of this sensitized state. It is initiated when activated sensory terminals release neuropeptides, stimulating local non-neuronal cells like mast cells and macrophages. This interaction establishes a self-amplifying feedback loop that primes the acupoint, enhancing its responsiveness to acupuncture. However, the precise mechanism through which visceral disease engages this neuroinflammatory cascade to modulate the acupoint's sensory interface remains undetermined. Peripheral sensory neurons, commonly referred to as nociceptors, are emerging as critical players for acupuncture manipulation. Typically, C-nociceptors, comprised of peptidergic and non-peptidergic subtypes, have testified the essential role in the onset of acupoint sensitization [14–18]. Among these, silent nociceptors, also referred to as mechanoinsensitive nociceptors (MINs), have been implicated in the initiation of pain onset and exacerbation [19–22], and itch [23]. These neurons, which innervate visceral organs and somatic tissues [19, 21, 24], are typically unresponsive to mechanical stimuli under normal conditions but become sensitized and mechanosensitive in the presence of inflammatory mediators, such as nerve growth factor (NGF). Recent study has identified the nicotinic acetylcholine receptor subunit alpha-3 (CHRNA3), a subunit of the nicotinic acetylcholine receptor, as a specific molecular marker of MINs in mice [20, 21]. CHRNA3 neurons densely innervate somatic tissues and viscera and account for 40% of the peptidergic C-nociceptors, contributes to secondary mechanical hyperalgesia development [20, 21]. Electrophysiological evidence reveals the “awakening” features of CHRNA3^+^ neurons, which convert from mechano-insensitive to mechano-responsive following NGF through TrkA/pERK/PIEZO2 signaling pathway. Given this mechano-responsiveness similarity, we propose that CHRNA3^+^ MIN mediate both the mechanical allodynia of sensitized acupoints and the amplification of sensory input during acupuncture.

In this study, we identify CHRNA3⁺ neurons as a distinct subset of C-fiber nociceptors in rats that innervate the colon and the BL25 acupoint via axonal bifurcation, providing a structural basis for viscerosomatic Integration. Using a colitis-induced acupoint sensitization model, we demonstrate that CHRNA3⁺ MINs are activated by colonic inflammation and contribute to colitis-induced referred mechanical hypersensitivity at the BL25 acupoint, a classical site used in acupuncture for gut-related dysfunctions. Chemogenetic inhibition of CHRNA3⁺ MINs not only alleviated acupoint hyperalgesia but also significantly reduced the efficacy of electroacupuncture (EA) in both behavioral and spinal neuronal readouts. These findings suggest that CHRNA3⁺ MINs not only mediate pathological sensitization but also act to prime the sensory interface. This process likely occurs through facilitating nociceptor-derived neuropeptide-mediated neurogenic inflammation and a localized neuroimmune cascade, which heightens mechanosensitivity and responsiveness to EA via the recruitment of the mechanosensitive PIEZO2 channel. Our study offers a mechanistic basis for how CHRNA3^+^ MINs drive acupoint functional plasticity to enhance acupuncture’s modulatory effects.

## Materials and methods

### Animals

Male Sprague-Dawley rats (220 ± 10 g) were purchased from Charles River (Beijing; license: SCXK-[Beijing]−2021-0011) and housed at the Institute of Acupuncture and Moxibustion, China Academy of Chinese Medical Sciences (CACMS). Rats were maintained under standard laboratory conditions (12 h light/dark cycle, 24 ± 0.5 °C, 60–70% humidity) and allowed to acclimate for 1 week before experimentation. All procedures were approved by the Institutional Animal Care and Use Committee of CACMS (Ethics No. Y2022-03–10-06).

### TNBS-induced colitis model

Colitis was induced as previously described [25]. After a 24-h fast, rats were anesthetized with 2% isoflurane, and TNBS (100 mg/kg in 50% ethanol, 2:1 ratio, Sigma-Aldrich) was administered intracolonically via a 30-gauge catheter inserted 6–7 cm from the anus. Rats were held vertically for 5 min post-injection to ensure retention. Control rats received equal volumes of saline or 50% ethanol. Animals were monitored daily for body weight, general condition, and signs of colitis.

### Disease activity index (DAI) and Tissue damage index (TDI)

Colitis severity was assessed using the DAI and TDI scoring systems [26]. DAI was calculated as the average of three parameters: weight loss (0–4), stool consistency (0 = normal, 2 = loose, 4 = diarrhea), and rectal bleeding (0 = none, 2 = occult, 4 = gross). TDI was assessed on H&E-stained colon sections by two blinded pathologists, evaluating inflammatory infiltration, crypt loss, ulceration, and mucosal damage. Images were captured using a microscope (BX53, Olympus, Tokyo, Japan) and evaluated with Image J software (US National Institutes of Health, Bethesda, MD).

### Evans blue plasma extravasation

Skin plasma protein extravasation following Evans Blue (EB) administration in colitis rats was conducted as previously described [27]. Briefly, rats were anesthetized with 2% isoflurane at 7 days after colitis induction, and Evans Blue (50 mg/kg in saline, Sigma) was injected into the lateral tail vein. After 30–60 min, PE points were mapped and counted. Hair was removed from the dorsal and hindlimb regions 24 h prior using depilatory cream (CP-8000, Codos, China).

### Mechanical sensitivity testing

Mechanical thresholds at BL25 were measured using an electronic von Frey apparatus (ALMEMO 2450, AHLBORN) as previously described [27]. Rats were acclimated to a restraining pocket for 2 days and habituated for 30 min before each test. Measurements were taken on days 1, 3, and 7, and the mean of three trials was used.

### AAV virus injection

To investigate whether dorsal root ganglion (DRG) neurons innervate both the colon and the BL25 acupoint region via axonal bifurcation, scAAV2/R-hSyn-EGFP (2.0 × 10^12^ VG/mL, PT-2315, BrainVTA) and scAAV2/R-hSyn-mCherry (2.0 × 10^12^ VG/mL, PT-3975, BrainVTA) were separately injected into the colonic wall and BL25 region. For intracolonic injections, a 1 cm midline abdominal incision was made in rats under isoflurane, and 10 µL of EGFP virus was injected between the muscularis externa and serosa at 5–6 evenly spaced sites (~ 2.5 cm total length) using a microsyringe under microscopic guidance. This multi-site, low-volume-per-site strategy was designed to maximize coverage while minimizing local tissue stress. For intracutaneous injections at BL25, a total of 10 µL of mCherry virus was delivered across 5–8 evenly distributed points in the bilateral BL25 region. Ten days after injection, L6–S1 DRGs were harvested, sectioned, and processed for colocalization analysis.

To selectively inhibit colonic CHRNA3⁺ neurons, rAAV-Chrna3-ZsGreen (2.6 × 10^12^ VG/mL) and rAAV-Chrna3-hM4D(Gi)-ZsGreen (Braincase) were injected into the colonic wall of the rat. A 1 cm incision was made under isoflurane anesthesia, and 10 µL virus was injected between the muscularis externa and serosa across 5–6 sites (~ 2.5 cm span). Colitis modeling was conducted 21 days after AAV injection to allow for sufficient transgene expression. Behavioral and electrophysiological experiments were conducted 7 days after colitis modeling (28 days after virus injection). The timeline was consistent for all chemogenetic experiments.

### Immunofluorescence and in situ hybridization

Rats were perfused with PBS followed by 4% paraformaldehyde. DRG, skin, colon, and spinal tissues were harvested, cryoprotected in 30% sucrose, embedded in OCT, and cryosectioned at 20 µm. Sections were blocked with 10% serum and incubated overnight at 4 °C with primary antibodies, followed by fluorescent secondary antibodies and DAPI. Confocal imaging was performed using an FV1000 microscope (Olympus, Tokyo, Japan). The following primary antibodies were used: CHRNA3, (Invitrogen, PA5-77501, 1:200), PIEZO2 (Novus, NBP2-58161, 1:50), TrkA (R&D, AF1056, 1:200), TrkA (NOVUS, AF1494, 1:200), p-ERK (Santa Cruz, sc-7383, 1:200), NeuN (Sigma, MAB377, 1:200).

RNAscope Multiplex Fluorescent Reagent Kit V2 (Cat. No. 323110) was used to perform in situ hybridization with *chrna3* (527051-C1, ACD) and *Piezo2* (549741-C3, ACD) probes according to the manufacturer’s protocol.

### Western blot assay

Seven days after colitis modeling, the bilateral lumbar L6-S1 DRG was collected and homogenized in ice-cold RIPA lysis buffer (89900, Thermo Scientific) with Halt Protease Phosphatase Inhibitor (5872 s, CST, 1:500). Protein concentrations were measured with a Pierce BCA Protein Assay Kit (23225, Thermo Scientific). Equal amounts of the proteins sample were resolved on 10% sodium dodecyl sulfate–polyacrylamide gel electrophoresis (SDS-PAGE) and then transferred to polyvinylidene difluoride membranes. Proteins samples were then incubated with antibodies for rabbit anti-ERK1/2 (A4782, Abclonal, 1:2000), rabbit anti-pERK1/2 (AP0472, Abclonal, 1:1000), rabbit anti-NGF (Abcam, Abclonal, ab52918, 1:2000), and β-actin (XAb-A, X-blot) overnight at 4 ℃, followed by incubation with the corresponding secondary antibodies. The interested bands were visualized using an ECL kit (170–5060, BIO-RAD) and imaged with iBright1500 (Invitrogen). The β-actin served as the loading control and intensity of the selected bands was analyzed using Image J.

### U0126 treatment

U0126 (5 mg/kg/day, i.p.) was administered to rat for 7 days after colitis induction. Stock solution (50 mg/mL in DMSO) was diluted to 5 mg/mL in 10% DMSO, 40% PEG300, 5% Tween-80, and 45% saline.

### Electroacupuncture (EA)

Rats were placed in a prone position on a heating pad and anesthetized with inhalation of 2% isoflurane. The bilateral BL25 acupoints are located at the depression lateral to the lower border of the spinous process of the fourth lumbar vertebra [28]. The needles (0.25 × 25 mm, Suzhou Medical Appliance Factory, Suzhou, China) were inserted into the bilateral BL25 point at a depth of ~ 3 mm. EA was delivered using a stimulator (STG4000, Warner Instruments, U.S.A) with the following parameters [26]: 2 Hz frequency, 1 mA intensity, for a duration of 10 min.

### Visceromotor reflex (VMR) recording

Visceromotor response (VMR) was recorded as an indicator of visceral hyperalgesia (VH) by colorectal distension (CRD) which was evoked by using a custom-made balloon [26]. After rats were anesthetized with 2% isoflurane, a lubricated polyethylene balloon (1 cm diameter) was inserted transanally into the colorectum approximately 0.5–1 cm from the anal verge. The external portion was secured to the tail with tape. Colorectal distension was produced by rapidly inflating the balloon to a constant pressure of 60 mmHg. The pressure was maintained for 20 s and allowed 5 min for recovery between each inflation. EMG spikes were recorded by the PowerLab Data Recording & Analysis System (ADIstruments Pty Ltd).

### Spinal LFP recording

The experimental setup for in vivo spinal LFP recording was similar to our previous studies^51^. Briefly, the rats were anesthetized with urethane (1.2 g/kg, i.p.). The lumbar spinal cord was exposed in anesthetized rats and the dura mater was partially removed at the recording segments (L6-S1). The paralyn-coated tungsten microelectrode (3 mΩ, Frederick Haer Company, Brunswick, ME, USA) was inserted into the superficial dorsal horn (200–500 μm below the surface) at L6 spinal segment. The left sciatic nerve was exposed and placed on the hook electrode to deliver the test stimulus (5 mA, 0.2 ms, 1 test/1 min), evoking the spinal LFP. A real-time, computer-based data acquisition and processing system (CED Spike 2, Cambridge, UK) was used to collect analog data. Raw data was collected at a sampling rate of 1000 Hz. The data stream was amplified and then filtered (0.1–100 Hz, model DAM80; World Precision Instruments, Sarasota, FL, USA), and artifacts of stimulation were removed online by a notch filter (IIR filters of the CED 1401 data-acquisition system).

LFP was examined before EA (baseline, 10 min) and at 0–30 min after EA application. Based on the conduction velocity, LFPs that correspond to activated A- and C-fibers can be distinguished. In comparison to the large A-LFP, the C-LFP exhibits a longer latency (90–130 ms) and smaller amplitude. Offline modulus measurements of Spike2 software were used to analyze the area under curves (AUC) of C-LFP. A comparison was made between pre- and post-EA conditions within and between the groups after normalizing LFP to pre-EA baseline values in each animal.

### Statistical analysis

Sample sizes were determined based on established standards in the field for similar models and endpoints, which consistently yield large effect sizes. This approach aligns with the ethical goal of minimizing animal use while ensuring robust detection of biological effects. For behavioral and functional assays (e.g., VMR and spinal LFP recording), n = 6–8 was used. For immunofluorescence and WB quantification, a smaller group size of n = 3–4 was employed, as these assays also yield large effect sizes with low variability in our experimental model, making this number both scientifically adequate and consistent with ethical reduction principles. The GraphPad Prism (version 8.0, GraphPad Software, San Diego, CA, USA) software was used for statistical analysis. The normality of data was checked using the Shapiro‒Wilk test. Data are presented as mean ± SEM. Paired or independent t-test was used to analyze data within two groups. One-way or two-way ANOVA with post hoc Bonferroni test was utilized for group comparison. Significance was set at *P* < 0.05.

## Results

### CHRNA3⁺ nociceptors innervate peripheral tissues and prime acupoints for colitis-induced sensitization

**Fig. 1:**
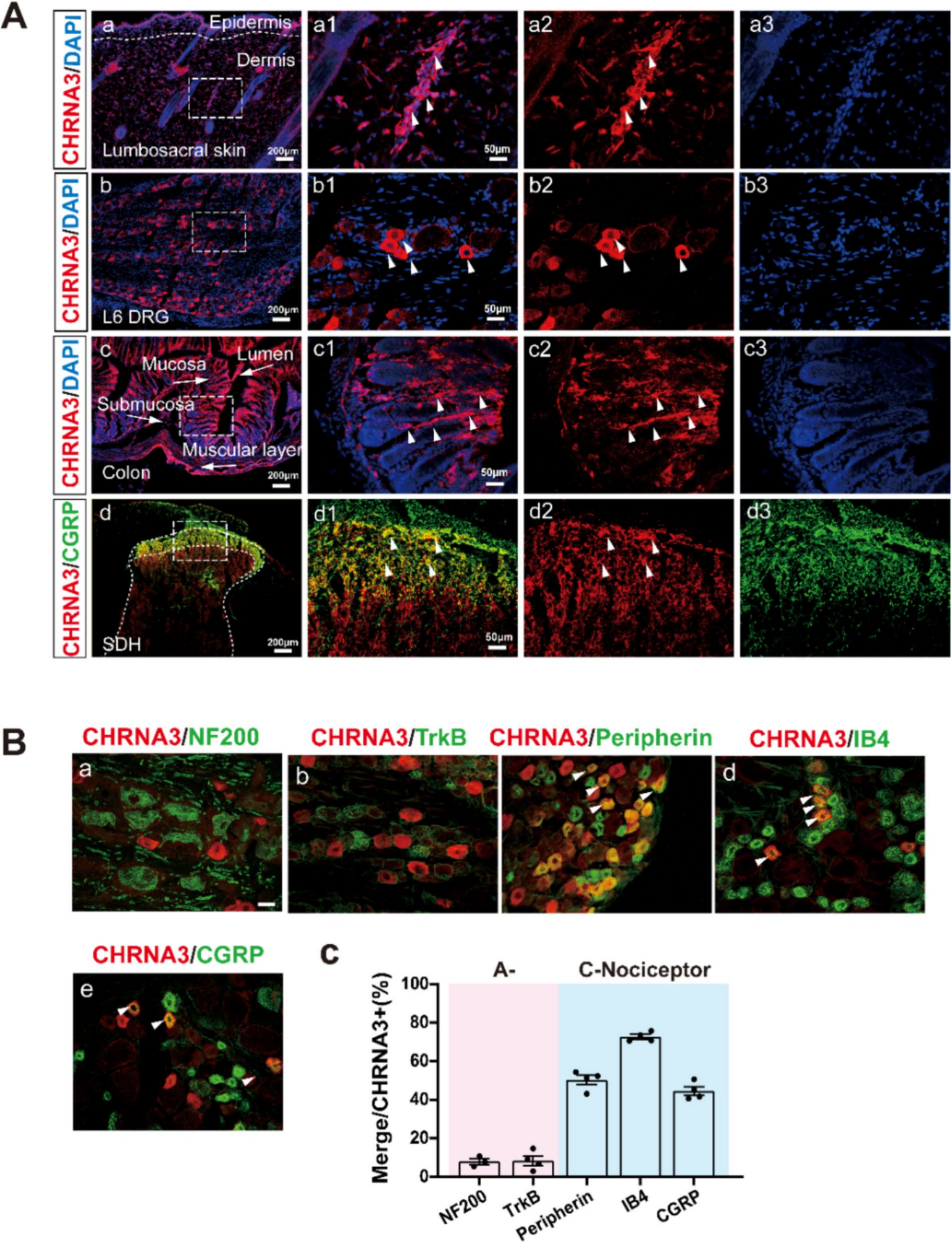
CHNRA3 + labeled mechanoinsensitive nociceptors (MINs) innervate peripheral tissue. **Aa**–**d** Immunostaining of tissue sections showing CHRNA3⁺ sensory fibers and neurons (red, white arrows) in the skin (**a**), L6 DRG (**b**), Colon (**c**), and spinal cord (**d**). Magnified views of dashed white boxes are shown in **a1**–**a3**, **b1**–**b3**, **c1**–**c3**. **B** Neurochemical profiling of CHRNA3⁺ nociceptors (red) by co-staining with A-nociceptor markers NF200 (**a**), TrkB (**b**) and C-nociceptor markers peripherin (**c**), non-peptidergic IB4 (**d**), and peptidergic CGRP (**e**) in rat DRG. Scale bar, 50 μm. **C** Proportion of co-localized neurons within the total CHRNA3 + population

Concerning previous studies of CHRNA3 neurons in pain conducted in mice, we herein examined the innervation patterns of CHRNA3 + mechanoinsensitive nociceptors (MINs) in rats for the first time. Using immunofluorescence, we observed that CHRNA3⁺ MINs innervate the lumbar skin, colon, L6 dorsal root ganglion (DRG), and lamina I of the L6–S1 spinal dorsal horn (Fig. 1A), indicating their widespread peripheral and central connectivity.

To determine the nociceptor subtype identity of CHRNA3⁺ neurons, we performed co-staining with markers for A-fiber neurons (NF200, TrkB), C-fiber markers (Peripherin), and subtype indicators for peptidergic (CGRP⁺) and non-peptidergic (IB4⁺) populations. CHRNA3⁺ neurons showed minimal overlap with A-fiber markers (NF200: 7.94%; TrkB: 8.37%) but significant co-expression with C-fiber markers (Peripherin: 50.29%), IB4 (72.69%), and CGRP (44.55%) (Fig. 1B, C). These data confirm that CHRNA3 is predominantly expressed in C-fiber nociceptors, covering both peptidergic and non-peptidergic subtypes.

### CHRNA3⁺ MINs mediate colitis-induced acupoint sensitization

**Fig. 2:**
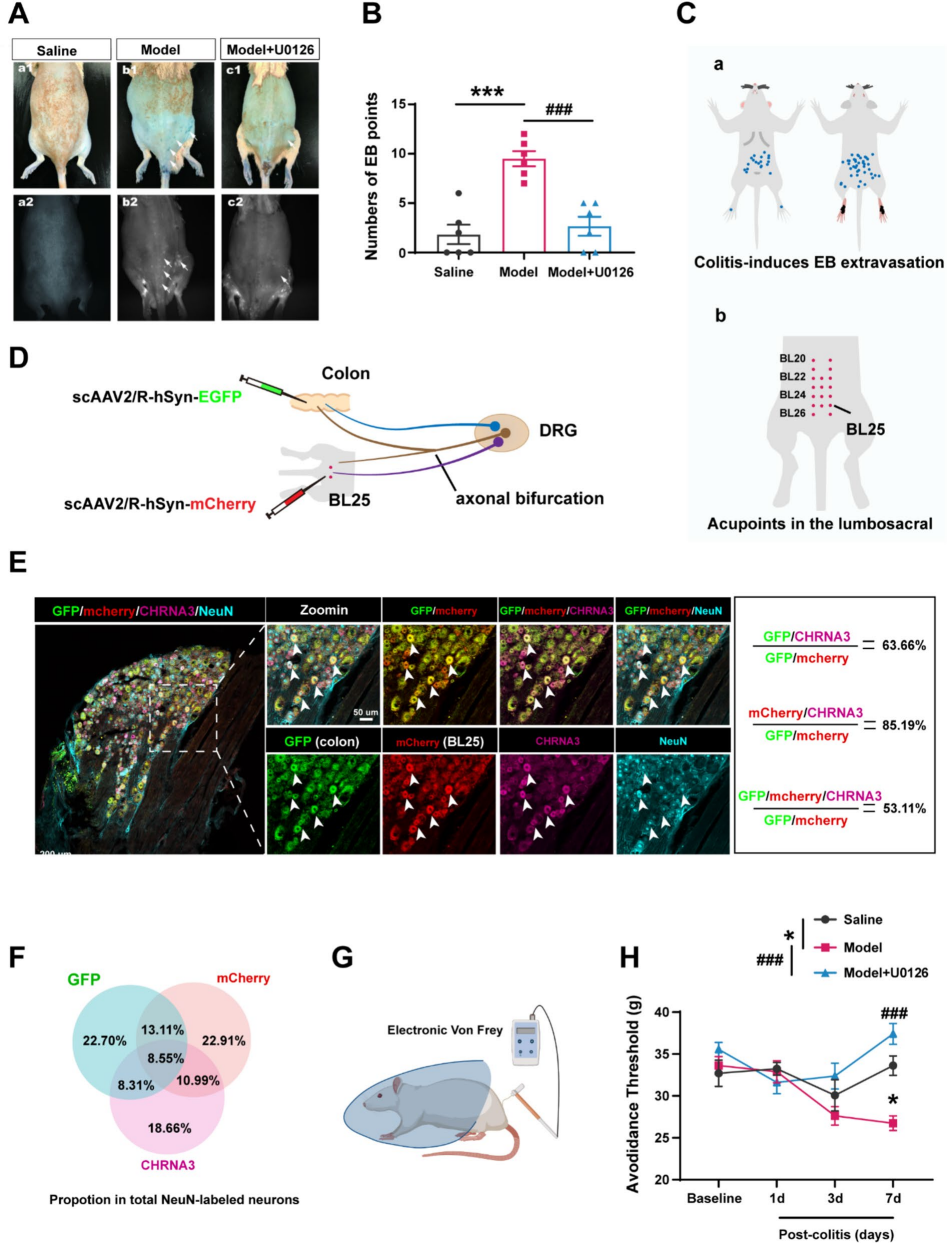
Blockade of CHRNA3 + nociceptor activation attenuated colitis-induced acupoint sensitization. **A** Representative images showing plasma extravasation (PE) points in the back 7 days after colitis induction. The extravasation of blue plasma proteins was validated using an in vivo imaging system (white arrow). **B** Quantification of PE points. ***P < 0.001vs. saline; ###*P* < 0.001 vs. Model, n = 6 per group. One-way ANOVA with Bonferroni test. **C** Mapping of overlapped PE points (**a**) coinciding with classical acupoint locations (**b**). **D** Schematic of scAAV2/R-hSyn-EGFP and scAAV2/R-hSyn-mCherry injections into the colon and BL25, respectively. **E** Representative images showing co-labeling of GFP⁺ colon-related neurons and mCherry⁺ BL25-related neurons with CHRNA3 (magenta) and NeuN (cyan) in L6 DRG. **F** Quantification of triple-labeled neurons (GFP⁺/mCherry⁺/CHRNA3⁺) among NeuN⁺ neurons. **G** Schematic diagram of mechanical threshold testing at the lumbosacral region. **H** Mechanical thresholds at BL25 before and 1, 3, 7 days after TNBS modeling. n = 7 for saline, 8 for Model and Model + U0126 group. *P < 0.05, Model vs. Saline; ### *P*< 0.001 Model vs. Model + U0126. Two-way repeated measures ANOVA with Bonferroni post hoc test

To assess the functional involvement of CHRNA3⁺ MINs in acupoint sensitization, we employed a TNBS-induced colitis model known to elicit referred somatic hypersensitivity [9, 29]. Evans blue (EB) dye was injected intravenously to visualize sites of neurogenic inflammation, marked by plasma extravasation (PE) points [30, 31]. Colitis significantly increased the number of PE points in the abdominal and lumbar regions compared to saline controls (Fig. 2A–C), and these overlapped with classical acupoints such as BL25 (Fig. 2C-b), consistent with previous studies about the sensitized acupoints under colitis.

Inhibition of CHRNA3⁺ MINs with U0126, a pERK inhibitor, significantly reduced the number of PE points. Given that mechanical hypersensitivity is a hallmark of sensitized acupoints, colitis-induced sensitized acupoint BL25 was selected and mechanical thresholds were assessed using electronic von Frey testing. Colitis significantly reduced mechanical thresholds at BL25, indicating mechanical allodynia, which was ameliorated by U0126 treatment (Fig. 2E, F). These findings suggest that activation of CHRNA3⁺ MINs is necessary for the onset of acupoint hypersensitivity and mechanical sensitization during colitis.

To investigate whether axonal bifurcation of CHRNA3⁺ neurons mediate the interaction between the colon and the BL25 acupoint region, scAAV2/9-hSyn-EGFP and scAAV2/9-hSyn-mCherry were injected separately into the colonic wall and the BL25 region, respectively. Co-staining with CHRNA3 and NeuN revealed that approximately 8.55% of total NeuN⁺ DRG neurons were triple-labeled with GFP, mCherry, and CHRNA3 (Fig. 2D, E). These findings indicate that a subset of CHRNA3⁺ neurons simultaneously innervate both the colon and the skin, providing a neuroanatomical basis for viscerosomatic crosstalk.

Additionally, we examined whether CHRNA3⁺ MINs contribute to tissue injury during colitis. TNBS significantly increased the colonic tissue damage index (TDI) and disease activity index (DAI) (Supplementary Fig. 1). Interestingly, U0126 reduced TDI scores but not DAI, implying that CHRNA3⁺ MINs may selectively modulate local tissue injury responses without altering overall disease burden.

### CHRNA3⁺ MIN activation is driven by TrkA/pERK/PIEZO2 signaling

**Fig. 3:**
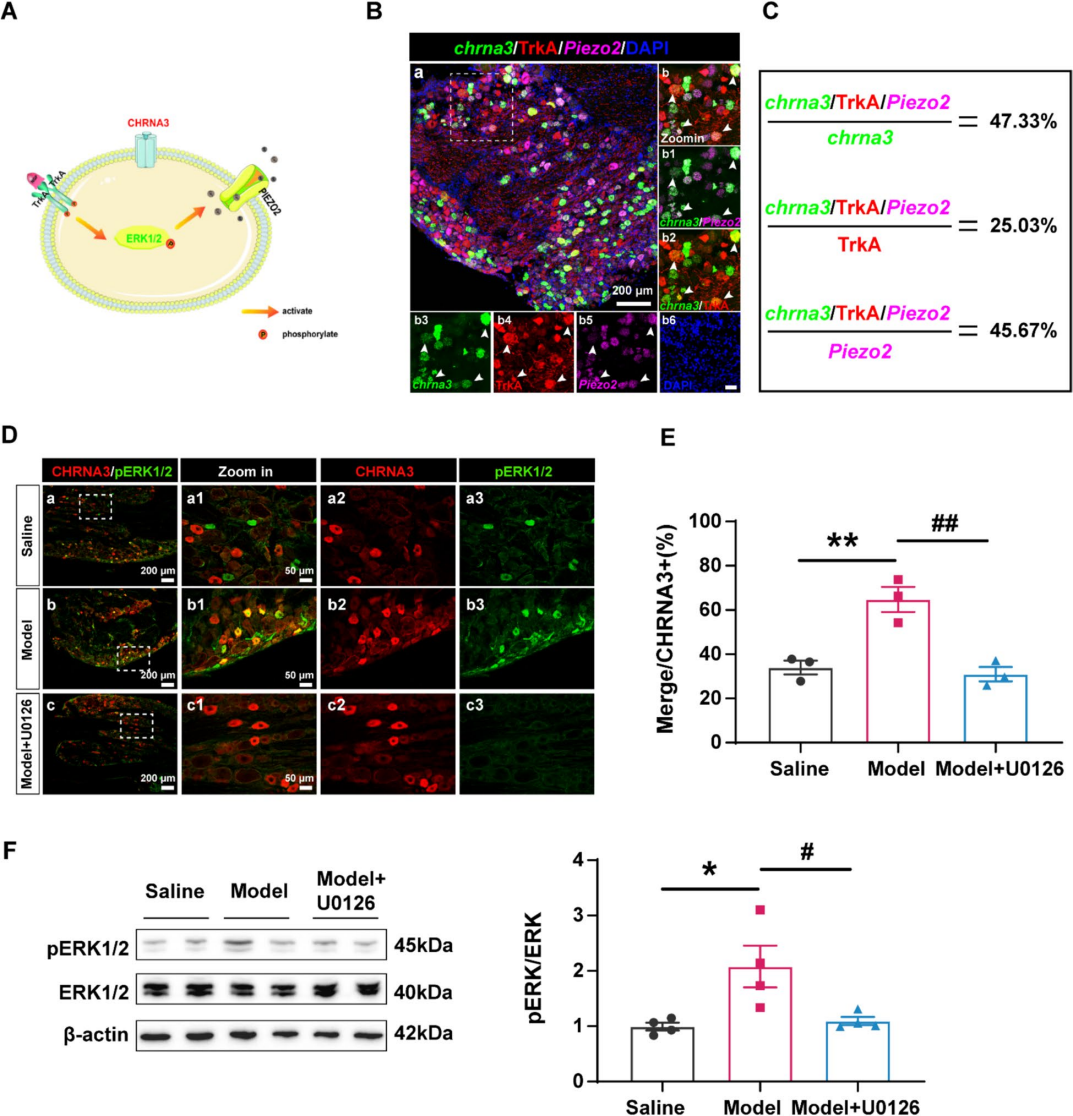
Colitis induces CHRNA3⁺ MIN activation via ERK1/2 signaling in L6 DRG. **A** Schematic illustrating intracellular signaling pathways for CHRNA3⁺ MIN activation. **B** Representative in situ hybridization images of L6 DRG co-stained for *chrna3* (green), *Piezo2* (magenta), and TrkA (red). Scale bar, 50 μm. **C** Quantification of triple-labeled (*chrna3*⁺/TrkA⁺/*Piezo2*⁺) neurons. **D** Representative immunostaining images of L6 DRG from Saline (**a**), Model (**b**), and Model + U0126 (**c**) groups stained for CHRNA3 (red) and pERK (green). Higher magnifications shown in **a1**–**a3**, **b1**–**b3**, **c1**–**c3**. **E** Quantification of CHRNA3⁺/pERK⁺ neurons among CHRNA3⁺ neurons. **P < 0.01vs. Saline; ##*P* < 0.01 vs. Model, n = 3 per group. One-way ANOVA with the Bonferroni test was used. **F** Representative immunoblotting images and quantification of protein levels of ERK1/2 and pERK1/2 in DRG of the Saline (**a**), Model (**b**), and Model + U0126 group. *P < 0.05 vs. Saline; #*P* < 0.05 vs. Model, n = 4 per group. One-way ANOVA with the Bonferroni test was used

Previous work in mice identified TrkA/pERK/PIEZO2 signaling as a key pathway underlying silent nociceptor activation [21]. Using *in situ* hybridization and immunofluorescence in rat L6 DRG, we confirmed that 47.33% of *chrna3*⁺ neurons co-expressed both TrkA and *Piezo2*, while 25.03% of TrkA⁺ and 45.67% of *Piezo2*⁺ neurons were triple-labeled (Fig. 3B, C; Supplementary Figure S2).

**Fig. 4:**
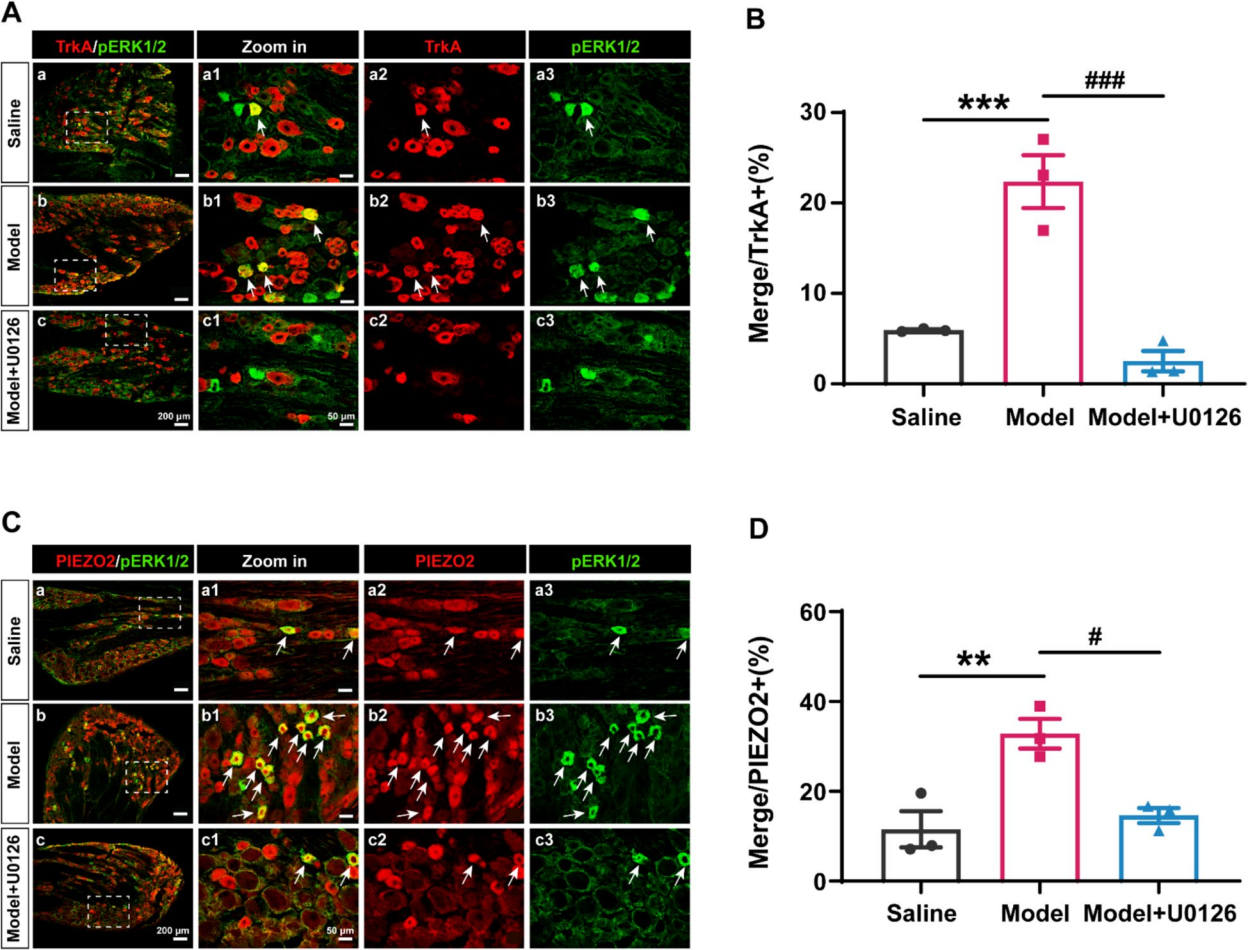
Colitis activates TrkA⁺ and PIEZO2⁺ neurons in L6 DRG. **A** Representative images of TrkA⁺ (red) and pERK⁺ (green) neurons in Saline (**a**), Model (**b**), and Model + U0126 (**c**) groups; magnified views in **a1**–**a3**, **b1**–**b3**, **c1**–**c3**. **B** Quantification of TrkA⁺/pERK⁺ neurons among total TrkA⁺ neurons. **P < 0.01, vs. Saline; ###p < 0.001 vs. Model, n = 3 per group. One-way ANOVA with the Bonferroni test. **C** Representative images of PIEZO2⁺ (red) and pERK⁺ (green) neurons; magnified views in **a1**–**a3**, **b1**–**b3**, **c1**–**c3**. **D** Quantification of PIEZO2⁺/pERK⁺ neurons in total PIEZO2 + neurons. *P < 0.05, vs. Saline; #*P* < 0.05 vs. Model, n = 3 per group. One-way ANOVA with the Bonferroni test

We next investigated whether colitis activates CHRNA3⁺ MINs through this pathway. Immunostaining showed a marked increase in pERK⁺ CHRNA3⁺ neurons in the DRG of colitis rats compared to saline controls (Fig. 3D, E). U0126 treatment significantly reduced this activation. Similarly, pERK co-localization with TrkA⁺ and PIEZO2⁺ neurons increased after colitis and was suppressed by U0126 (Fig. 4). To determine whether NGF is a potential upstream driver [21], we assessed NGF expression in the colon, DRG, and BL25 skin. NGF was significantly upregulated in colon and DRG, as well as an elevation trend in skin following colitis (Supplementary Fig. 2), supporting a role for NGF–TrkA–pERK–PIEZO2 signaling in CHRNA3⁺ MIN activation. These data strongly support a mechanism in which colitis activates CHRNA3⁺ MINs via NGF-dependent signaling, promoting their conversion from a mechanoinsensitive to a mechanoresponsive state and subsequently priming the acupoint for heightened sensory input.

### Chemogenetic inhibition of CHRNA3⁺ MINs alleviates acupoint hyperalgesia

**Fig. 5:**
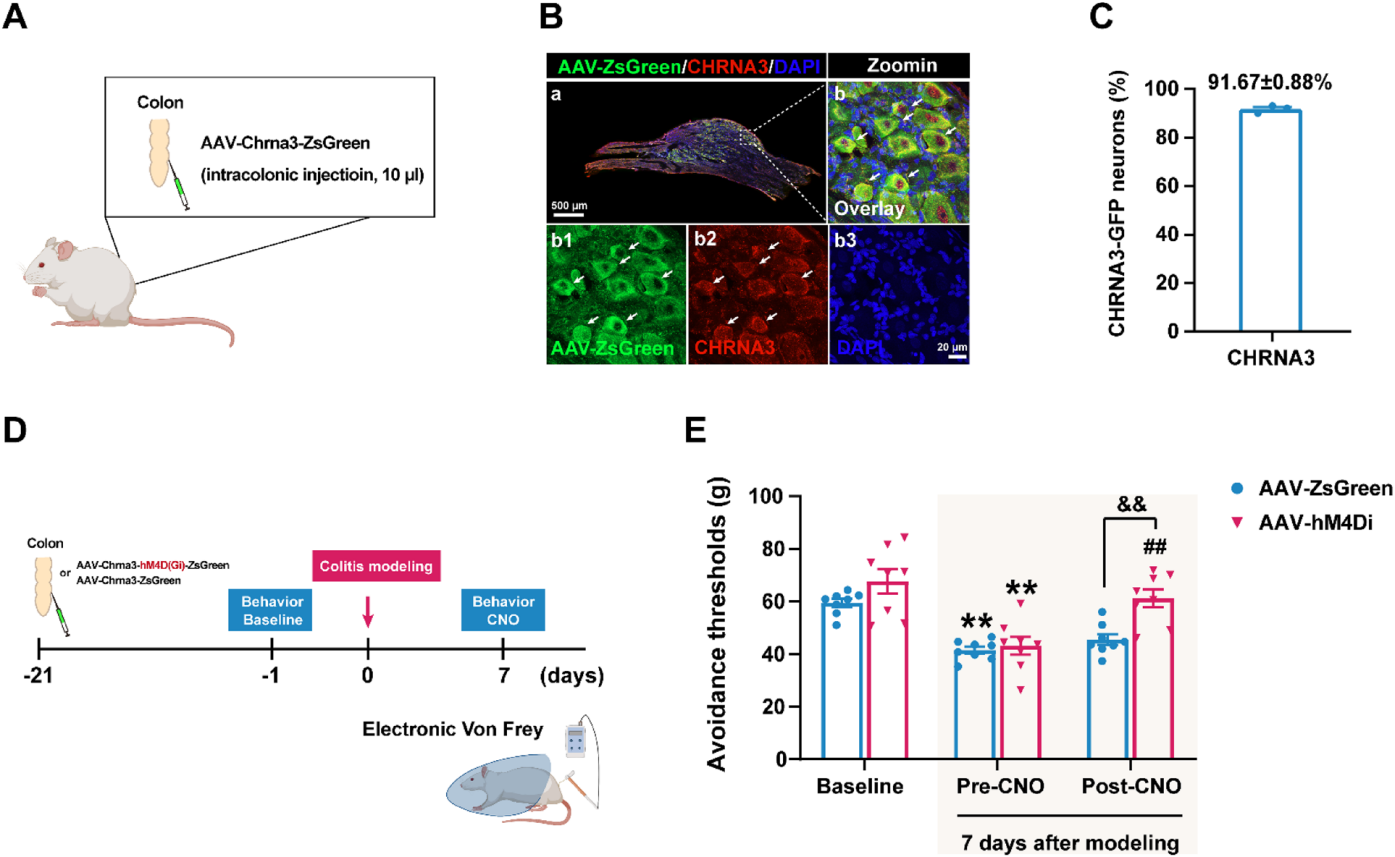
Chemogenetic inhibition of CHRNA3⁺ MINs mitigates colitis-induced mechanical hypersensitivity at BL25. **A** Schematic of intracolonic injection of AAV virus. **B** Representative images of ZsGreen-labeled neurons co-stained with CHRNA3 (red) and DAPI; magnified in **b1**–**b3**. **C** Quantification of ZsGreen⁺/CHRNA3⁺ neurons among CHRNA3⁺ neurons. N = 3. **D** Timeline of AAV-DREADD intervention and behavior testing. Chemogenetic inhibitory AAV-Chrna3-hM4Di-ZsGreen (AAV-hM4Di) and control virus AAV-Chrna3-ZsGreen (AAV-ZsGreen) were intracolonic injected in rats. Colitis modeling was conducted 21 days after virus injection to allow for sufficient transfection. Behavior tests were conducted before and 7 days after modeling, as well as the CNO administration. **E** Mechanical thresholds at BL25 pre- and post-TNBS modeling and pre- and post-CNO administration. N = 8 per group. **P < 0.01, compared to baseline; ##*P* < 0.01, compared to pre-CNO; &&*P* < 0.01, compared to pre-CNO, compared to AAV-ZsGreen. Two-way repeated measure ANOVA with Bonferroni post hoc test

To directly assess the contribution of CHRNA3⁺ MINs to mechanical hypersensitivity, we constructed an AAV vector expressing inhibitory DREADD (hM4Di) under the *Chrna3* promoter (AAV-Chrna3-hM4Di-ZsGreen) and confirmed that ~ 91.67% of CHRNA3⁺ neurons expressed the reporter 3 weeks post-intracolonic injection (Fig. 5A–C). CNO administration in AAV-hM4Di-transduced rats significantly increased mechanical thresholds at BL25 compared to pre-CNO and control virus (AAV-ZsGreen) animals, indicating attenuation of colitis-induced mechanical hypersensitivity (Fig. 5D, E). These results functionally confirm that CHRNA3⁺ MIN plays a role in impacting acupoint microenvironment, which then primed the interface and sustained the sensitized acupoint state for pathological mechanical responses.

### CHRNA3⁺ MINs facilitate electroacupuncture-induced analgesia

**Fig. 6:**
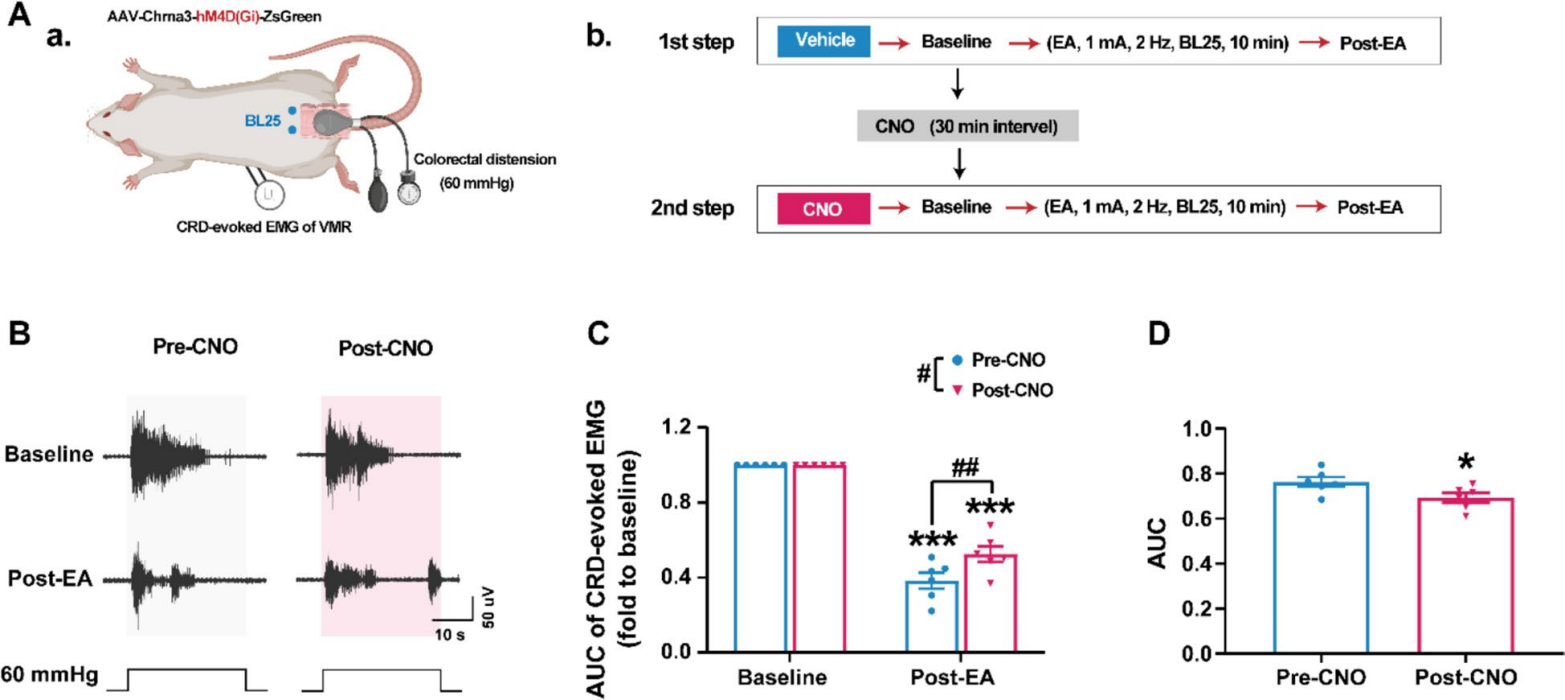
Chemogenetic silencing of CHRNA3 + MIN attenuates EA at BL25-induced analgesia on visceral hyperalgesia. **A** Experimental schematic of CRD-evoked VMR and EA protocol. **B** Representative traces of CRD-evoked VMR EMG upon EA stimulation (BL 25, 1 mA, 2 Hz, 10 min), Pre- and Post-CNO administration. **C** Fold change of area under the curve (normalized to baseline) of CRD-evoked VMR EMG before and post-EA intervention. n = 6. ***P < 0.001, compared to Baseline; #*P* < 0.05, ##*P* < 0.01, compared to Pre-CNO. Two-way repeated measure ANOVA with Bonferroni post hoc test. **D** Area under the curve shows the calculated change of the CRD-evoked EMG after EA intervention pre and post CNO administration. Paired t-test. *P < 0.05, compared to Pre-CNO

To determine whether CHRNA3⁺ MINs enhance acupuncture signaling, we assessed the analgesic effects of electroacupuncture (EA) at BL25 on colorectal distension (CRD)-evoked visceral motor reflexes (VMRs) [26]. EA significantly reduced the EMG area under the curve (AUC) during CRD in colitis rats, while CNO-induced silencing of CHRNA3⁺ neurons attenuated this inhibitory effect (Fig. 6A–C), suggesting that CHRNA3⁺ MINs activation may heighten BL25 responsiveness to facilitate EA-induced analgesia.

**Fig. 7:**
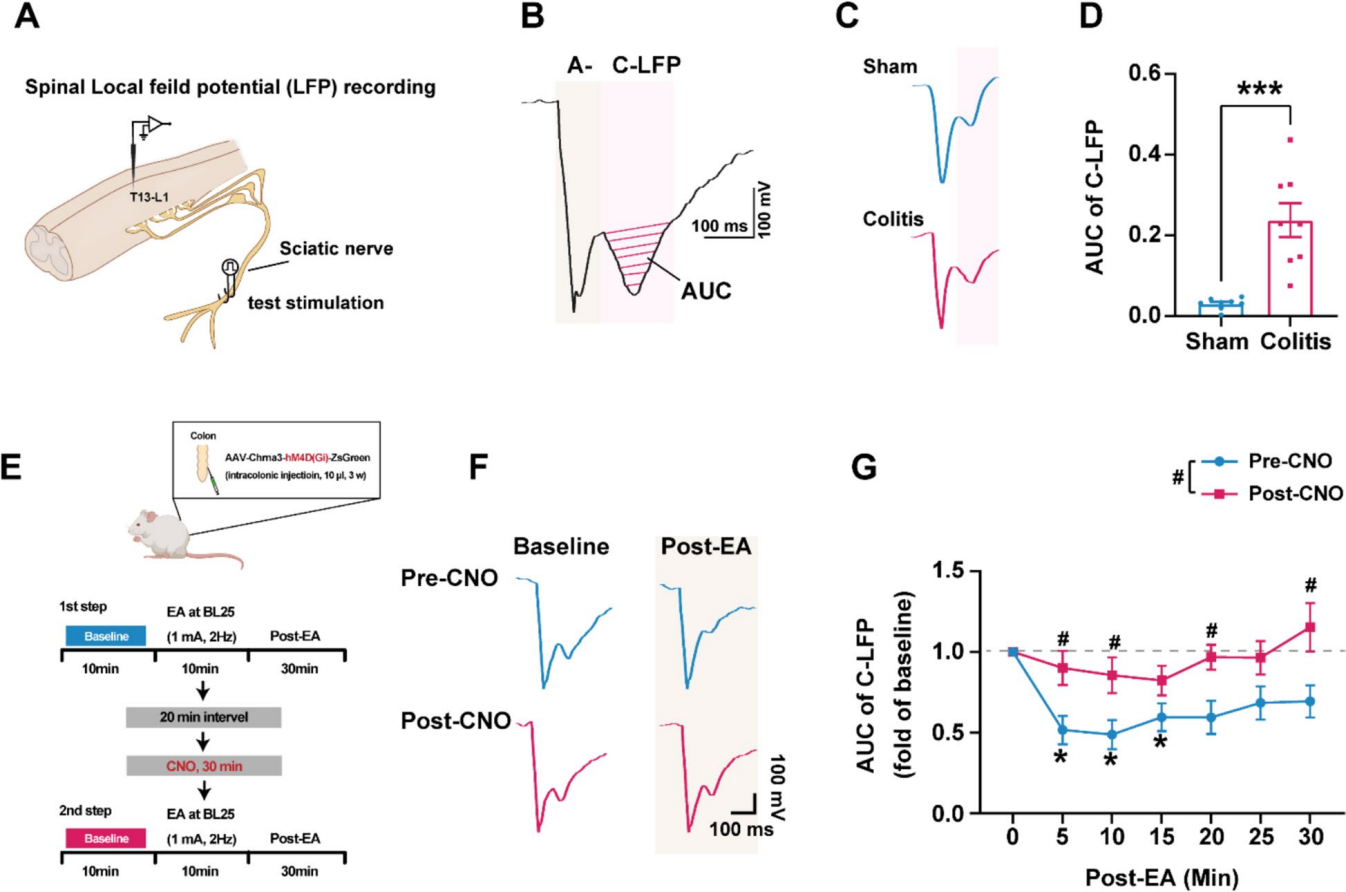
Activation of CHRNA3 + MIN potentiates EA-induced inhibition of spinal C-local field potentials (C-LFPs) in colitis rats. **A** Schematic diagram of in vivo spinal LFP recording setup. **B** Representative trace for sciatic nerve stimulation-evoked LFP to A-fiber inputs (A-LFP) and C-fiber inputs (C-LFP). **C** Representative traces of evoked LFP in sham and colitis group. **D** The areas under C-LFP curves of sham and colitis groups were plotted. N = 8 per group. ***P < 0.001, vs. Sham. Independent t-test. **E** Experimental protocol for EA modulation of C-LFPs pre- and post-CNO in AAV-Chrna3-hM4Di-ZsGreen rats. **F** Representative trace of LFP before and after EA (BL 25, 1 mA, 2 Hz, 10 min) pre- and post-CNO administration. **G** The AUC of C-LFP during each 5-min period after EA were averaged for analysis. N = 8. *P < 0.05, compared to baseline (time 0); #*P* < 0.05, compared to Pre-CNO; Two-way repeated measure ANOVA with Bonferroni post hoc test

To evaluate central effects, we recorded spinal cord C-local field potentials (C-LFPs) evoked by sciatic nerve stimulation. Colitis elevated C-LFP amplitudes compared to Sham, reflecting spinal sensitization [26]. EA at BL25 suppressed C-LFPs at 5, 10, and 15 min, but this suppression was abolished by CHRNA3⁺ MINs silencing (Fig. 7F, G). Together, these data indicates that CHNRA3^+^ MIN is involved in acupuncture’s signals transduction, suggesting CHRNA3⁺ MINs not only primes peripheral acupoints but also enables EA-driven inhibition of spinal nociceptive transmission.

## Discussion

**Fig. 8:**
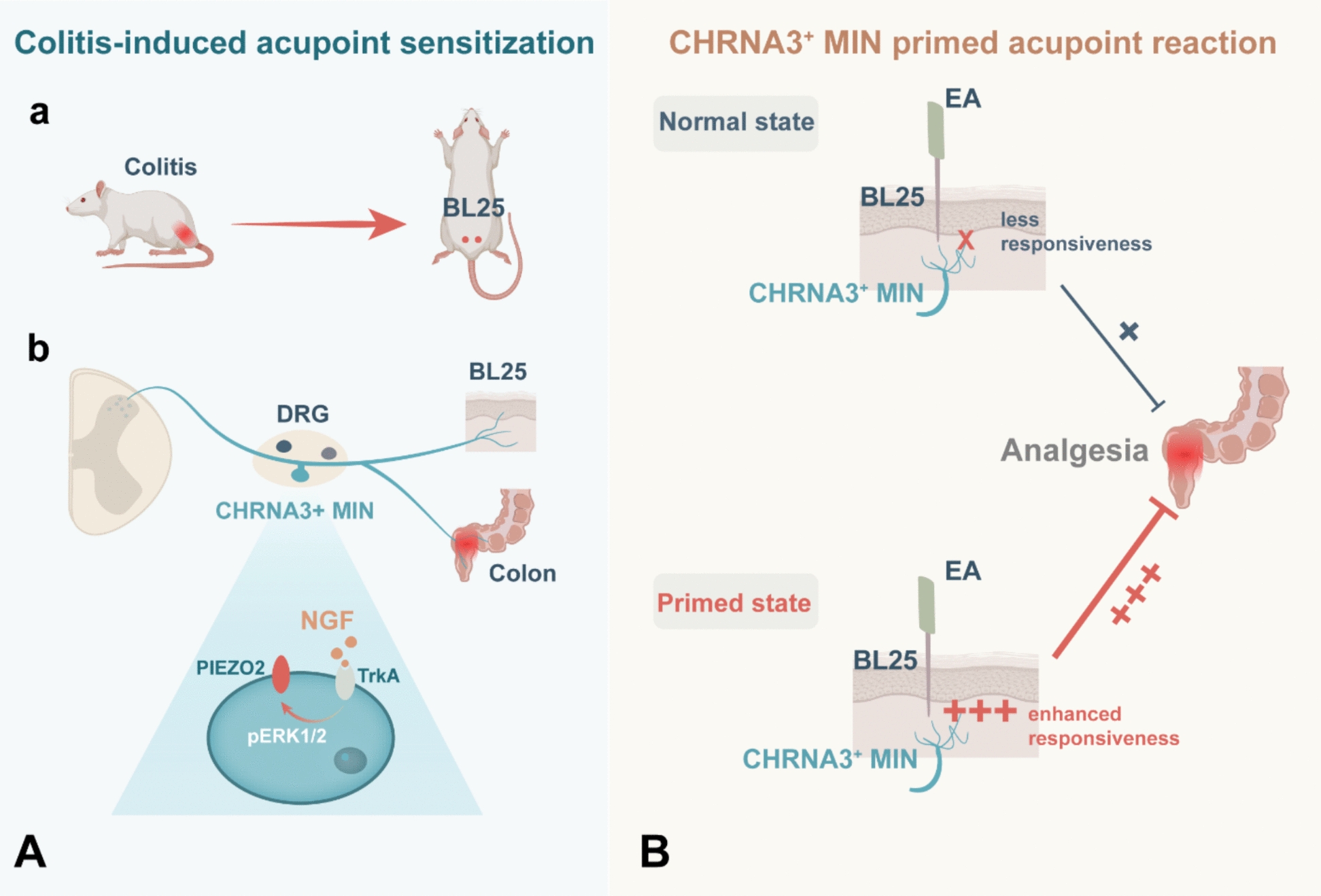
Schematic diagram summarizing how CHRNA3⁺ MINs prime acupoint responsiveness and enhance acupuncture efficacy in colitis. **A** Colitis leads to sensitization of acupoint BL25 (**a**) via axonal bifurcation of DRG neurons. Inflammatory components such NGF activate CHRNA3 + neurons, a subpopulation of C-nociceptors, through the TrkA/pERK/PIEZO2 signaling cascade (**b**). This converts CHRNA3^+^ neurons from mechanoinsensitive (silent) to mechanosensitive (awakened) state, contributing to colitis-induced mechanical hypersensitivity at BL25. **B** Awakened CHRNA3⁺ MINs primed the sensory interface of BL25 and mediated sensitization. This heightened responsiveness enhances the acupoint's reaction to EA stimulation and pontentiates EA analgesia for colitis-related visceral pain

Increasing evidence suggests that acupoints are associated with disease-induced referred somatic pain, a phenomenon known as acupoint sensitization [14, 32, 33]. However, the neural basis of acupoint sensitization, and how it mediates and transmits acupuncture signals, remains poorly understood. In this study, we identified CHRNA3, a known marker of mechanoinsensitive nociceptors (MINs) in mice, as being predominantly expressed in C-fiber nociceptors in rats, with widespread innervation of peripheral tissues. We further demonstrated that activation of CHRNA3⁺ MINs contributes to colitis-induced acupoint sensitization and mechanical hypersensitivity at the BL25 acupoint via the TrkA/pERK/PIEZO2 signaling pathway. Notably, these neurons appear to prime the peripheral sensory field, enhancing the responsiveness of acupoints to both nociceptive and therapeutic stimuli, such as EA. At the same time, colitis-driven CHRNA3⁺ MIN activation reshapes the acupoint sensory interface, converting it into a hyperexcitable state that facilitates signal amplification. Accordingly, chemogenetic inhibition of CHRNA3⁺ MINs not only alleviated mechanical hyperalgesia at sensitized BL25 but also attenuated the analgesic effects of EA on colitis-related visceral hypersensitivity. These findings highlight a dual role for CHRNA3⁺ MINs in priming and reshaping acupoint function, thereby modulating both pathological sensitization and acupuncture-induced analgesia (Fig. 8).

### Nociceptors mediate acupoint sensitization

The concept of acupoint sensitization has gained attention in recent decades, supported by clinical and preclinical observations that many acupoints overlap with regions of referred somatic hypersensitivity associated with visceral disease or tissue injury. Sensory hypersensitivity and functional enhancement are hallmark features of this phenomenon. Notably, mechanical allodynia is considered the primary manifestation of acupoint sensitization [6–9], aligning with the classical concept of "selecting the painful point as the acupuncture point" described in the *Jing Jin* chapter of the *Miraculous Pivot*. In this study, we confirmed that regions of plasma extravasation overlapped with colitis-related acupoints, supporting the pathological relevance of referred hypersensitivity [9, 34]. Meanwhile, although both the colon and the BL25 acupoint are innervated by the same spinal segments (L6–S1), we herein demonstrate that axonal bifurcation may serve as a potential neuroanatomical basis for colitis-induced acupoint sensitization.

While multiple studies have explored anatomical substrates contributing to acupoint function, including sensory nerves [1], blood vessels, lymphatic vessels [2], connective tissues [3], tissue space areas [4], and mast cells [5], no single structure clearly distinguishes acupoints from adjacent non-acupoint regions. This has led to a shift in understanding acupoints not as fixed structures, but as primed sensory interfaces at the somatic area, dynamically modulated by pathophysiological states and capable of integrating acupuncture inputs [2, 35].

Acupoints contain a complex neuroimmune microenvironment involving nociceptors, immune cells, blood vessels, and autonomic terminals [14, 36]. Among these, nociceptors act as the primary transducers of acupuncture-derived mechanical signals, engaging in acupoint sensitization and EA-induced neuromodulatory effects [14–16]. Recent transcriptomic advances, including single-cell RNA sequencing, have identified at least 18 distinct clusters of C-fiber nociceptors [37], broadly categorized into peptidergic (e.g., CGRP⁺, SP⁺) and non-peptidergic (e.g., IB4⁺) subtypes. We observed that CHRNA3 is predominantly co-expressed with peripherin and colocalizes with both peptidergic and non-peptidergic C-nociceptors (Fig. 1B, C). Previous studies have attributed acupoint sensitization to the heightened excitability and *I*_*h*_-current density in C-nociceptors, rather than Aδ-nociceptors [17]. The pathological profile of these sensitized sites is frequently defined by neurogenic inflammation, marked by the accumulation of nociceptor-derived neuropeptides such as CGRP and SP and inflammatory cascade [30, 31, 38]. Our data expand this mechanistic framework by identified CHRNA3⁺ MINs as essential initiator of acupoint sensitization. While only 40% of CHRNA3^+^ MINs co-localize with CGRP^+^ nociceptors, chemogenetic inhibition of these neurons significantly attenuated sensory hypersensitivity of acupoint. These findings suggest that colitis-awakened CHRNA3^+^ MINs may prime BL25 acupoint sensory interface, potentially by promoting CGRP release and augmenting neurogenic inflammation via axonal bifurcation; Furthermore, since nociceptor-derived CGRP and SP are established mediators of sensory-immune crosstalk, CHRNA3^+^  MINs-derived CGRP and SP likely exacerbate the local neuroimmune response. Collectively, these mechanisms converge to reshape the acupoint sensory interface, heightening responsiveness to diverse external stimuli. The precise molecular signaling downstream of these interactions warrants further investigation.

### Silent nociceptor activation primes the acupoint sensory interface

C-nociceptors are further distinguished by their responsiveness to thermal, chemical, or mechanical stimuli, which may shift under pathological conditions [39–42]. Silent nociceptors, also referred to mechanoinsensitive nociceptors, represent a unique subtype that are typically unresponsive to mechanical stimuli under normal conditions but acquire mechanosensitivity following inflammation or injury [21]. This "awakening" process is well documented across species: approximately 15–20% of cutaneous C-fibers in humans are silent nociceptors, with even higher proportions (30–90%) reported in visceral and articular tissues in rodents [19, 21, 43–45]. In a mouse model of colitis, the proportion of mechanosensitive C-fibers rose from 34 to 53%, while silent nociceptors decreased from 27 to 13%, implying their conversion to an active state. Recent work also highlights the role of MINs in secondary mechanical hypersensitivity in joint inflammation [20].

The Lechner group identified CHRNA3 as a reliable molecular marker for MINs in mice, demonstrating that their acquisition of mechano-responsiveness is driven by the NGF/TrkA/pERK signaling cascade which subsequently recruits mechanical channel PIEZO2 [21]. Further investigations identified TMEM100 as a key downstream molecular switch regulating this “awakening” and mediating secondary hyperalgesia [20]. Based on these insights, we propose that CHRNA3⁺ MINs exert a dual role in facilitating hypersensitivity of sensitized acupoints and heightening acupuncture signals input. Consistent with this model, our data confirmed co-expression of CHRNA3 with TrkA, pERK, and PIEZO2 in rat DRG neurons and demonstrated that CHRNA3⁺ MINs are activated by colitis. Interestingly, our data revealed a significant elevation of NGF levels in the colon and DRG, whereas only a non-significant upward trend was observed in the skin. This spatial heterogeneity suggests that while colonic NGF release is directly triggered by TNBS-induced inflammation at the primary site of pathology, the sensitization of the referred BL25 acupoint is driven by secondary neurogenic mechanisms. Although the modest elevation of cutaneous NGF may still be functionally relevant via combining with other bioactivitors, we propose that the substantial NGF accumulation within the colon and DRG serves as the primary initiator of sensitization, which likely awakens the dichotomized CHRNA3^+^ MINs to induce acupoint sensitization. This process is orchestrated via axonal bifurcation, likely in coordination with other neural mechanisms such as dorsal root reflex, viscera-somatic convergence and facilitation [14]. Furthermore, we confirmed the functional involvement of the NGF-TrkA/pERK/PIEZO2 axis in acupoint sensitization. This was evidenced by an increased co-expression portion of TrkA and PEIZO2 with pERK following TNBS administration, an effect that was reversed by the MEK inhibitor U0126. Chemogenetic silencing of these neurons abolished their mechanical conversion and reduced both acupoint sensitization and mechanical hypersensitivity. These findings suggest that the activation of PIEZO2 channel, driven by the NGF-TrkA/pERK cascade, is a primary driver of the heightened mechano-responsiveness observed at BL25. Ultimately, CHRNA3^+^ MINs facilitates a nociceptor-derived neuropeptide-mediated neuroimmune response, likely driven by the local release of CGRP and SP, that dynamically primes the acupoint microenvironment. By reshaping local sensory interface and lowering the threshold for mechano-responsiveness through recruiting the PIEZO2 channel, CHRNA3^+^ MINs promotes the acupoint from a “silent” region into “active” sensory hub. This transformation significantly potentiates the acupoint's responsiveness to acupuncture-derived mechanical stimuli and subsequent signal transduction.

Additionally, because we did not assess CHRNA3 expression in autonomic ganglia, including the pelvic ganglion, or in the nodose ganglion (which is critical for interoceptive signaling) during our chemogenetic viral experiments, the potential contributions of CHRNA3⁺ neurons in these ganglia remain unclear and warrant further investigation.

### CHRNA3⁺ MINs amplify acupuncture signaling

Functionally, CHRNA3⁺ MINs activation appears to enhance the analgesic effects of acupuncture. Silencing CHRNA3⁺ neurons attenuated EA-induced inhibition of visceral pain responses and spinal neuronal hyperactivation. Previous studies have shown that C-nociceptors are essential for acupuncture effects: for example, TRPV1 knockout mice exhibit impaired EA-induced analgesia [15], and *Prokr2* sensory neurons mediate EA-driven anti-inflammatory responses [18]. However, most prior work has focused on how somatic sensory input generates downstream effects. In contrast, our study reveals a novel mechanism whereby CHRNA3⁺ MINs prime the sensory interface, rendering acupoints more responsive to mechanical and EA stimulation. Clinical and preclinical evidence increasingly highlights the superior therapeutic effect when targeting sensitized acupoints across conditions such as the management of chronic musculoskeletal and non-musculoskeletal pain [10–12], bronchial asthma [46], and allergic rhinitis [47], but the mechanism remains elusive. Hence, this model expands the current understanding of how acupoint sensitization enhances the acupuncture effect, rather than acting as passive targets. Sensitized acupoints are dynamically reconfigured by the “awakening” of silent CHRNA3^+^ MINs. This activation transforms the acupoint into a functionally excitable zone by promoting neuropeptide release, facilitating neurogenic inflammation and neuroimmune crosstalk, and recruiting the mechanosensitive PIEZO2 channel. This reconfiguration potentiates the acupoint's responsiveness to acupuncture manipulation, facilitating high-efficiency signal transduction. Whether this priming is further driven by sensory neuronal coupling or localized axon reflexes remains a critical focus for future inquiry [14, 48–50].

## Conclusions

In summary, our findings suggest that CHRNA3⁺ MINs serve as key modulators in shaping acupoint sensory interface. Their colitis-driven conversion to a mechanosensitive state, a process consistent with the activation of the NGF–TrkA/pERK/PIEZO2 signaling axis, primes the sensory interface and enables the amplification of both pathological input and therapeutic signals. This reconfiguration positions CHRNA3⁺ MINs as a potential functional link connecting visceral disease-induced referral somatic hypersensitivity at the acupoint to the enhancement of acupuncture efficacy.

### Limitations of the study

While our findings establish a novel mechanism linking CHRNA3⁺ MINs to acupoint sensitization and analgesia, several limitations warrant consideration. First, our conclusions are derived primarily from a single visceral disease model (TNBS-induced colitis) and its segmentally related acupoint (BL25). Whether CHRNA3⁺ MINs mediate sensitization in other pathological states or at non-segmental or heterotopic acupoints (e.g., ST36) remains to be determined. Second, although we acknowledge the critical neuroimmune microenvironment of acupoints, this study focused on the peripheral neuronal component; the specific interactions between activated CHRNA3⁺ MINs and local immune cells (e.g., mast cells, macrophages) were not explored. Third, the potential presence and functional role of CHRNA3⁺ MINs in autonomic ganglia or vagal afferent neurons require further investigation. Finally, while our spinal C-LFP recordings demonstrate a transient analgesic effect of acute EA involving CHRNA3⁺ MINs, whether this pathway also contributes to the long-lasting or cumulative analgesic effects seen with repeated treatment, as is typical in clinical practice, remains to be determined. Furthermore, our study design does not delineate whether CHRNA3⁺ MINs contribute to the broader systemic effects of acupuncture, such as modulation of visceral function or psychological state. These open questions highlight important avenues for future research.

## Supplementary Information

Supplementary material 1: Figure 1 Blockade of CHRNA3+ nociceptor ameliorated TNBS-induced colitis, related to Figure2. A. Representative histopathologic images of colon tissue 7 days after modeling for each group. B. Comparison of tissue damage indexscores from the histopathologic images among groups. ***P＜0.001, compared to saline; ###*P*＜0.01, compared to Model, n=6 per group. One-way ANOVA with the Bonferroni test. C. The scores of disease activity indexof rats in the three groups were compared. ***P＜0.001, compared to saline, n=6 per group. One-way ANOVA with the Bonferroni test. Figure 2. Colitis increases NGF expression in colon, DRG, and BL25 skin. *P＜0.05, compared to Saline, n=3 per group; independent t-test.

Supplementary material 2.

## Data Availability

The data will be made available on reasonable request.

## Abbreviations

AUC: Area under the curve
CGRP: Calcitonin gene-related peptide
CHRNA3: Nicotinic acetylcholine receptor subunit alpha-3
CRD: Colorectal distension
DAI: Disease activity index
DRG: Dorsal root ganglion
EA: Electroacupuncture
EB: Evans blue
EMG: Electromyography
LFP: Local field potential
LTMR: Low-threshold mechanoreceptor
MIN: Mechanoinsensitive nociceptor
NGF: Nerve growth factor
SP: Substance P
TDI: Tissue damage index
TNBS: 2,4,6-Trinitrobenzenesulfonic acid
TrkA: Tropomyosin receptor kinase A (NGF receptor)
TRPV1: Transient receptor potential vanilloid 1
VMR: Visceromotor reflex
