# Supplementary material for: CHRNA3⁺ nociceptors prime the cutaneous sensory interface to enhance electroacupuncture analgesia
Source: Chin Med. 2026 May 19;21:134. doi: 10.1186/s13020-026-01425-w (PMC13185271; doi:10.1186/s13020-026-01425-w)
Supplement: Supplementary file 1 — Supplementary material 1: Figure 1 Blockade of CHRNA3+ nociceptor ameliorated TNBS-induced colitis, related to Figure2. A. Representative histopathologic images of colon tissue 7 days after modeling for each group. B. Comparison of tissue damage indexscores from the histopathologic images among groups. ***P＜0.001, compared to saline; ###P＜0.01, compared to Model, n=6 per group. One-way ANOVA with the Bonferroni test. C. The scores of disease activity indexof rats in the three groups were compared. ***P＜0.001, compared to saline, n=6 per group. One-way ANOVA with the Bonferroni test. Figure 2. Colitis increases NGF expression in colon, DRG, and BL25 skin. *P＜0.05, compared to Saline, n=3 per group; independent t-test. [file 13020_2026_1425_MOESM1_ESM.docx]

**
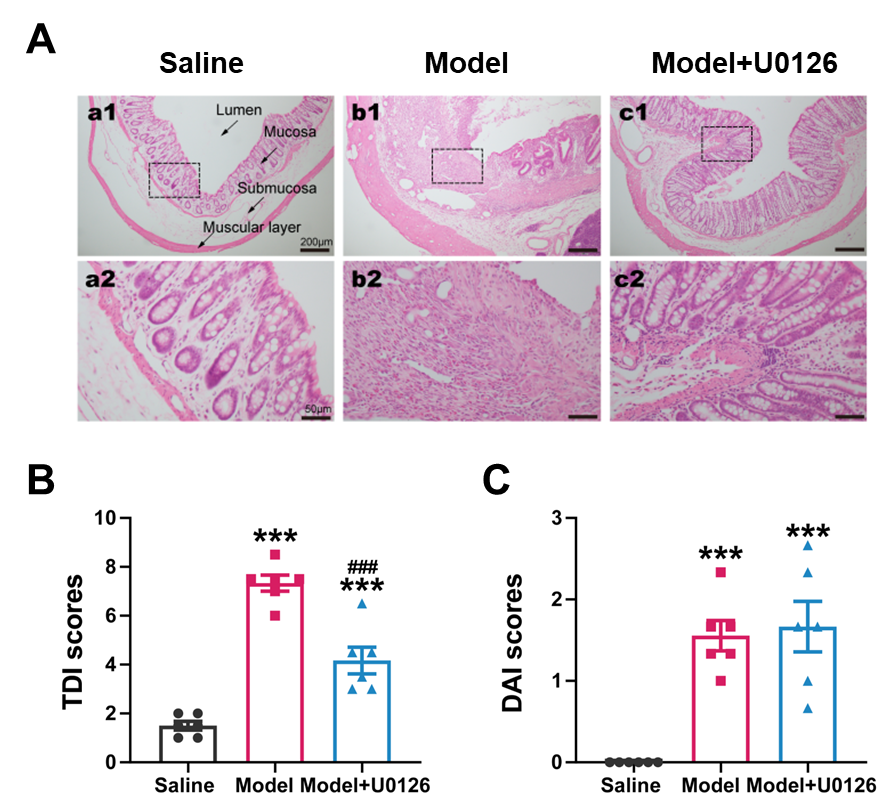
Supplementary**

**Supplementary Figure 1 Blockade of CHRNA3+ nociceptor ameliorated TNBS-induced colitis, related to Figure2.**

**A.** Representative histopathologic images of colon tissue 7 days after modeling for each group.

**B.** Comparison of tissue damage index (TDI) scores from the histopathologic images among groups. ***p＜0.001, compared to saline; ###p＜0.01, compared to Model, n=6 per group. One-way ANOVA with the Bonferroni test.

**C.** The scores of disease activity index (DAI) of rats in the three groups were compared. ***p＜0.001, compared to saline, n=6 per group. One-way ANOVA with the Bonferroni test.


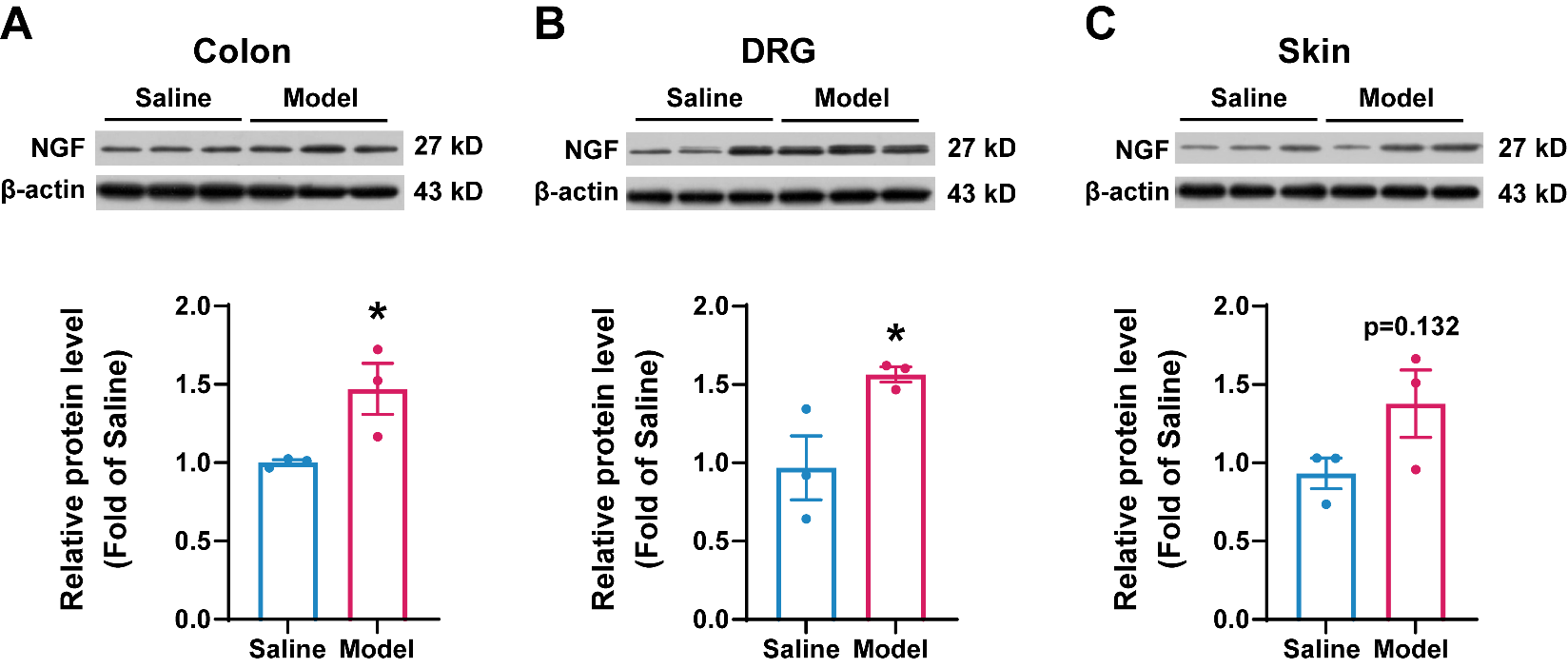


**Supplementary Figure 2. Colitis increases NGF expression in colon (A), DRG (B), and BL25 skin (C).** *P＜0.05, compared to Saline, n=3 per group; independent t-test.
