# Supplementary material for: CHRNA3⁺ nociceptors prime the cutaneous sensory interface to enhance electroacupuncture analgesia
Source: Chin Med. 2026 May 19;21:134. doi: 10.1186/s13020-026-01425-w (PMC13185271; doi:10.1186/s13020-026-01425-w)

**Supplymentary files, related to figure3 F**

The order of the sample is as follows: Saline, Model, Model+U0126, N=2 for each group per blot.

**1. pERK**

**
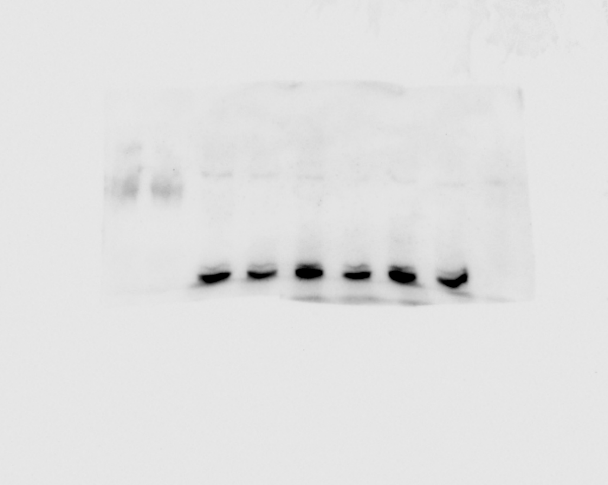
**
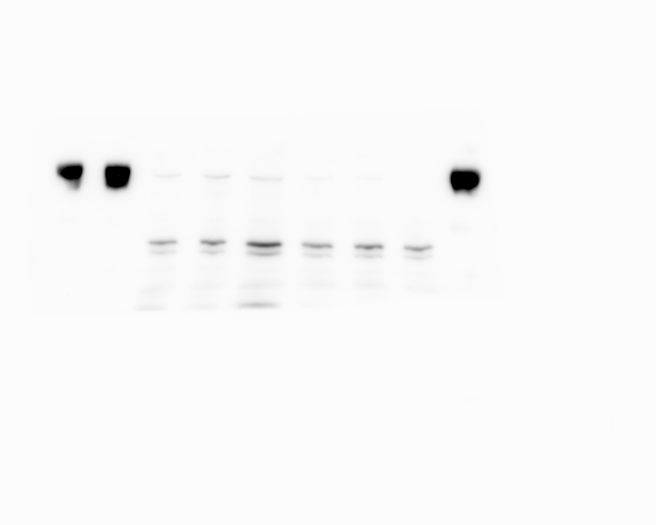


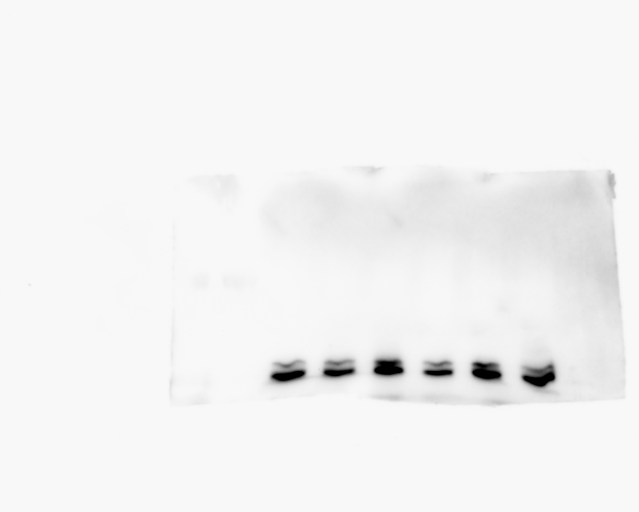

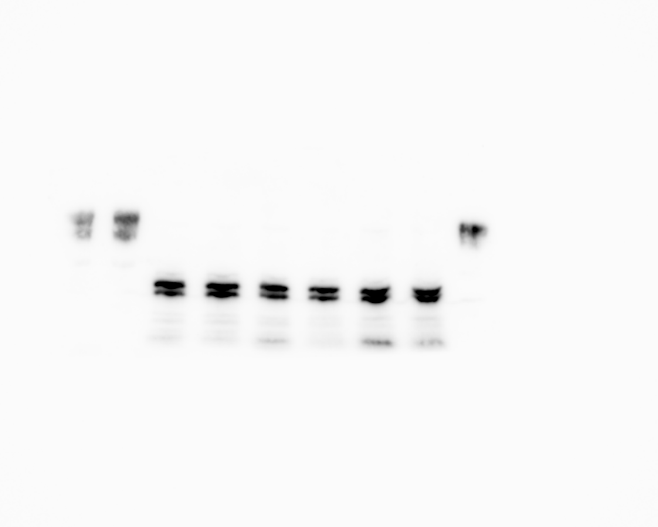
**2. ERK**

**3. β-Actin**


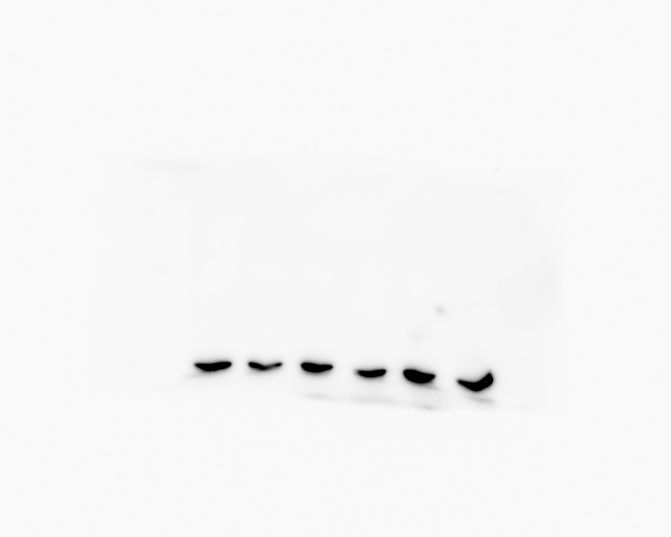

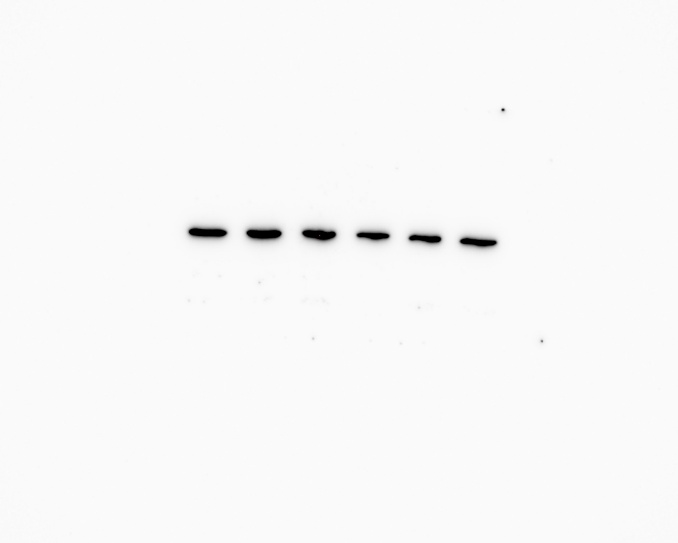

Supplement: Supplementary file 2 — Supplementary material 2. [file 13020_2026_1425_MOESM2_ESM.docx]
